# Temporal and Spatial Analysis of Clinical and Molecular Parameters in Dextran Sodium Sulfate Induced Colitis

**DOI:** 10.1371/journal.pone.0006073

**Published:** 2009-06-29

**Authors:** Yutao Yan, Vasantha Kolachala, Guillaume Dalmasso, Hang Nguyen, Hamed Laroui, Shanthi V. Sitaraman, Didier Merlin

**Affiliations:** Division of Digestive Diseases, Department of Medicine, Emory University School of Medicine, Atlanta, Georgia, United States of America; Charité-Universitätsmedizin Berlin, Germany

## Abstract

**Background:**

Inflammatory bowel diseases (IBD), including mainly ulcerative colitis (UC) and Crohn's disease (CD), are inflammatory disorders of the gastrointestinal tract caused by an interplay of genetic and environmental factors. Murine colitis model induced by Dextran Sulfate Sodium (DSS) is an animal model of IBD that is commonly used to address the pathogenesis of IBD as well as to test efficacy of therapies. In this study we systematically analyzed clinical parameters, histological changes, intestinal barrier properties and cytokine profile during the colitic and recovery phase.

**Methods:**

C57BL/6 mice were administered with 3.5% of DSS in drinking water for various times. Clinical and histological features were determined using standard criteria. Myeloperoxidase (MPO) activity, transepithelial permeability and proinflammatory mediators were determined in whole colon or proximal and distal parts of colon.

**Results:**

As expected after administration of DSS, mice manifest loss of body weight, shortening of colon length and bloody feces. Histological manifestations included shortening and loss of crypts, infiltration of lymphocytes and neutrophil, symptoms attenuated after DSS withdrawal. The MPO value, as inflammation indicator, also increases significantly at all periods of DSS treatment, and even after DSS withdrawal, it still held at very high levels. Trans-mucosal permeability increased during DSS treatment, but recovered to almost control level after DSS withdrawal. The production of proinflammatory mediators by colonic mucosa were enhanced during DSS treatment, and then recovered to pre-treated level after DSS withdrawal. Finally, enhanced expression of proinflammatory mediators also revealed a different profile feature in proximal and distal parts of the colon.

**Conclusion:**

Experimental colitis induced by DSS is a good animal model to study the mechanisms underlying the pathogenesis and intervention against IBD, especially UC.

## Introduction

Inflammatory bowel diseases (IBD), including mainly ulcerative colitis (UC) and Crohn's disease (CD), are inflammatory disorders of the gastrointestinal tract caused by multiple factors, including genetic and environmental factors, and are characterized by diarrhea, bloody stools, abdominal pain, and weight loss. Histological characteristics of IBD include crypt abscesses, crypt distortion and loss, ulcerationan infiltration of large numbers of neutrophils, monocytes, and lymphocytes. In past decades, dozens of different models of experimental IBD have been developed to investigate pathogenesis and improve treatment options. Although no model serves as a complete surrogate for IBD, many enable us to study the pathogenic characteristics of IBD, to identify novel genes that are possibly involved in disease susceptibility, and to characterize pivotal immunological molecules and processes. IBD experimental models can generally fall into five different categories: I) Gene knockout (KO) models: Interleukine (IL)-2/IL-2 receptor-alpha [1], IL-10 [2], T cell receptor (TCR) [3], trefoil factor [4], Tumor necrosis factor (TNF)-3′ untranslated region (UTR) [5]; II) Transgenic models: IL-17 [6], signal transducer and activating transcription (STAT)-4 [7], HLA B27 [8]; III) spontaneous colitis models: C3H/HejBir [9], SAMP1/YitFc mice [10]; IV) Inducible colitis models: Trinitrobenzene sulfonic acid (TNBS) colitis [11], [12], dextran sulfate sodium (DSS) colitis [13], peptidoglycan-polysaccharide (PG-PS) colitis [14] and V) Adoptive transfer models: heat shock protein (HSP) 60-specific CD8 T cells transfer induced colitis [15], CD45RB transfer model [16]. Most commonly, experimental colitis is induced by heparin-like polysaccharide DSS because of its simplicity and the high degree of uniformity and reproducibility of the colonic lesions [16]. By interfering with intestinal barrier function firstly and then stimulating local inflammation as the secondary phenomenon, DSS is often used to induce the mouse model of colitis which can mimic clinical and histoogical features of IBD with UC characteristics. Although the clinical and histological parameters are well established, the cytokine profile and its correlation with other parameters are unknown. The present work is a rigorous analysis of the general profile of experimental colitis induced by DSS, including the clinical, histological characteristics, as well as inflammatory indicators and mediators in a spatial and temporal fashion.

## Results

### Weight loss and colon length shortening during DSS treatment

**Figure 1:**
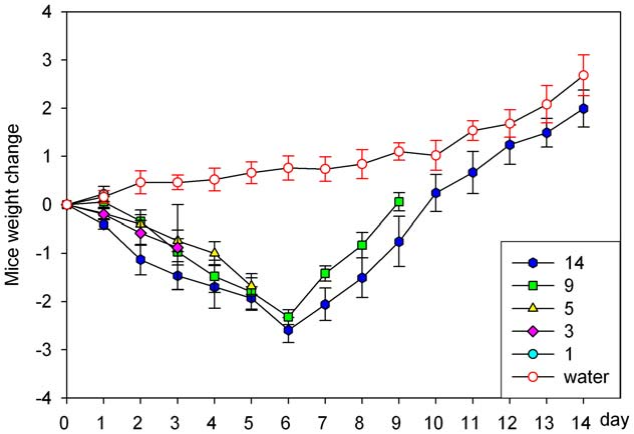
Mouse body weight changes during DSS treatment. C57BL/6 mice were administered to 3.5% DSS by drinking water for indicated days. Body weight changes are depicted as means±SEM body weight changes in each group. * P<0.05, ** P<0.01.

**Figure 2:**
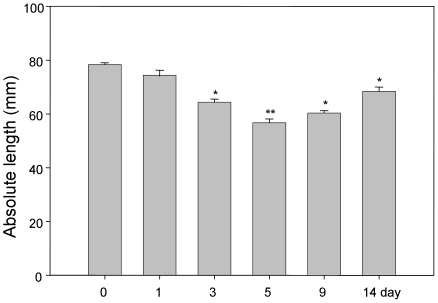
Mouse colon size changes during DSS treatment. C57BL/6 mice were exposed to 3.5% DSS in their drinking water for indicated days. Colon length removed from euthanized mice is depicted as means±SEM length of the colon in each group. * P<0.05, ** P<0.01.

We induced experimental colitis in C57BL/6 mice by adding 3.5% DSS to the drinking water for indicated days. DSS intake did not differ between the different groups of mice (data not shown). Mice showed different extent of diarrhea, more and grosser rectal bleeding as DSS treatment progressed, which suggested presence and development of inflammation. Specially, after 3-days of DSS treatment, more than 70% (17/24) of mice in 3-, 5-, 9- and 14-day treated groups had diarrhea and occult blood or gross blood in the feces, and these signs disappeared in part after DSS was withdrawn for 4 days (day 9) and complete after DSS withdrawal for 9 days (day 14). It has been pointed out that 2 (out of 6) mice in the 5-day DSS treatment group died after DSS withdrawal. During the period of DSS treatment, the weight loss was noticed after DSS administration under the indicated days. As shown in Fig. 1, 3-, and 5-day groups, body weight significantly decreased during the DSS-treatment, much lower than those in normal drinking water-treated mice. After DSS withdrawal, mouse body weight recovers gradually. Colon length was also measured to determine the severity of colitis. We found that DSS could lead to significant reduction of colon length during the treatment as described in Fig. 2. This colon length shortening became most severe in 5-day DSS-treated mice compared to drinking water mice colon (Fig. 2) with a reduction in colon length of around 30%. The reduction could be recovered after withdrawal of DSS, such as day 9 and day 14 groups. In general, the mice receiving DSS had a clinical disease activity score of 9.4±1.1 in 5-day group based on the score system established previously [17].

### Histological characterization

**Figure 3:**
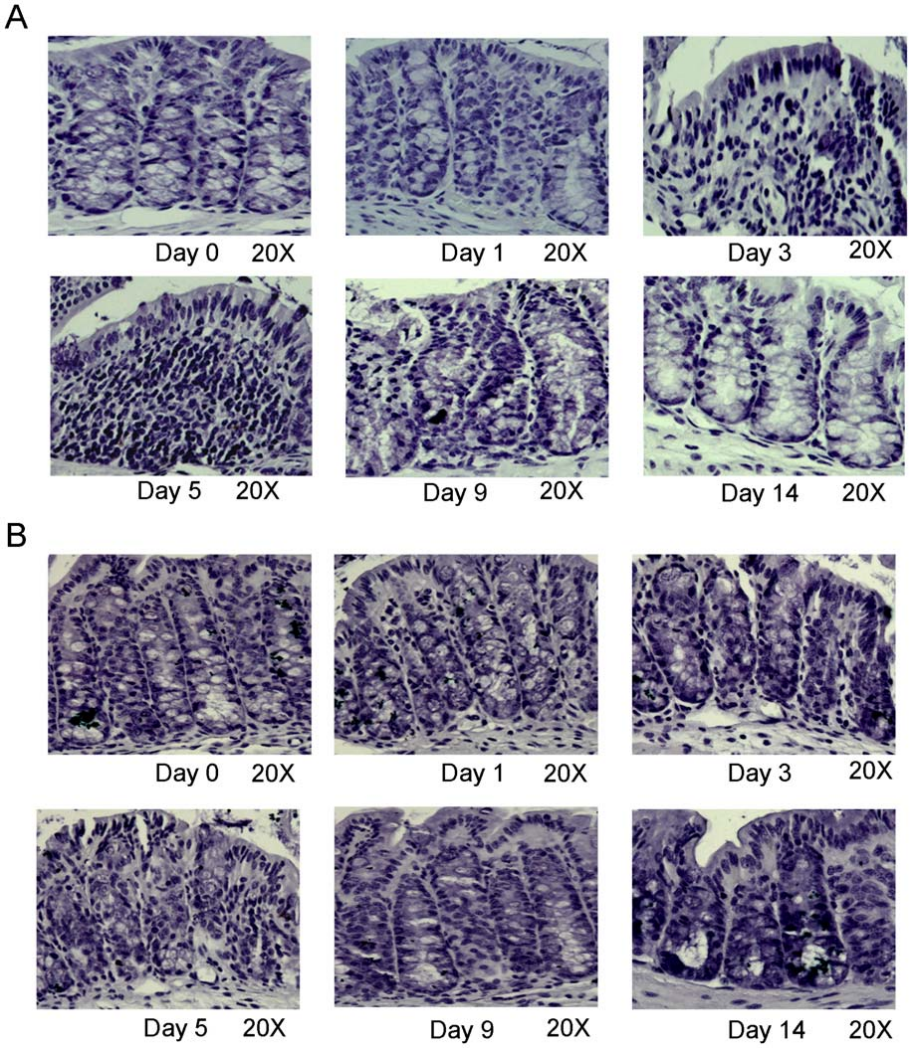
Hematoxylin-stained colon sections of mice treated with DSS at 0, 1, 3, 5, 9 (withdraw after 5 days treatment, 4 days to recover) and 14 (withdraw after 5 days treatment, 9 days to recover) days, with magnification of 20 times. (A): Distal colon; (B) Proximal colon.

Control mouse colon sections (Fig. 3, 0 day) showed the intact epithelium, well defined crypt length, and no edema neutrophil infiltration in mucosa and submucosa, and no ulcers or erosions. In contrast, colon tissue from DSS treated mice showed increasingly severe inflammatory lesions extensively throughout the mucosa during the DSS treatment (Fig. 3 1-, 3-, 5-day), then attenuating inflammatory lesions throughout the mucosa after DSS withdrawal. Ulcers, shortening and loss of crypts were seen focally at the beginning progressing to more extensive areas of mucosal involvement and finallythe whole colon (Fig. 3 1-, 3-, 5-day), which are in agreement with the data described previously [17], [18], then the mucosa were recovered to almost intact after DSS withdrawal (Fig. 3 9-, 14-day). Submucosal edema increased during DSS treatment 1-, 3-, 5-day group (Fig. 3) and recovered to normal state at day 9 and day 14 (Fig. 3); Infiltration of immune cells including neutrophils and lymphocytes were seen in the lamina propria in DSS treated mice (Fig. 3 1-, 3-, 5-day), and cleared after the DSS withdrawal in 9- and 14-day group.

### MPO activity

**Figure 4:**
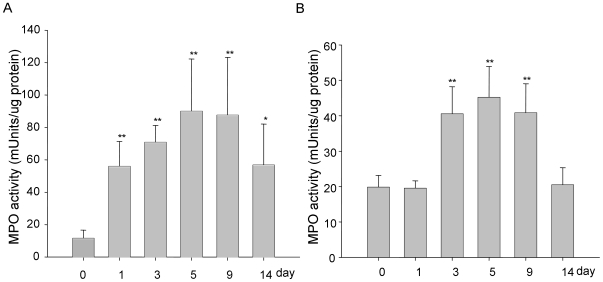
Determination of MPO enzymatic activity as an index of neutrophils infiltration into the injured tissue. (A): Distal colon; (B): Proximal colon. Results are expressed as MPO mUnits per µg protein and represent mean±SEM of 3 determinations. * P<0.05, ** P<0.01.

We measured colonic myeloperoxidase (MPO) activity as an indicator of the extent of neutrophil infiltration into the mucosa. MPO values were significantly higher in DSS-treated groups than corresponding controls at all the periods studied. Fig. 4 shows the quantification in concentrations of this parameter in the experimental animals. We found that DSS-induced increases of MPO activity reached to the maximum level of 92±28 mUnits/ug protein at day-5 group, 6-fold higher than that of the controls (14±3.5 mUnits/ug). After DSS was replaced with drinking water, MPO levels remained significantly higher at 9-day group (6-times increase as that in 5-day group). In the 14-day group, the MPO level had declined significantly but still higher than water-drinking group (Fig. 4).

### DSS treated mice exhibit epithelial barrier dysfunction

**Figure 5:**
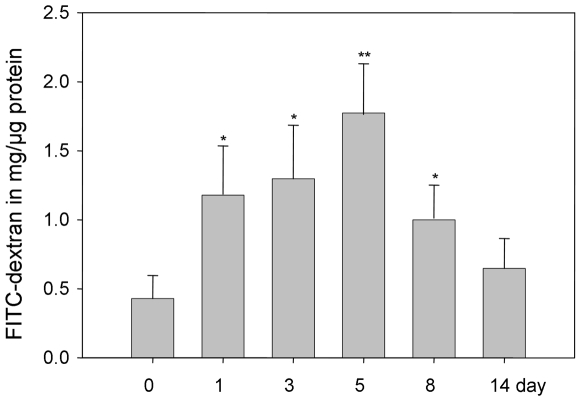
*In vivo* permeability assay in colon epithelium of mice treated with DSS. Results are presented as FITC-dextran in mg/µg protein and represent mean±SEM of 4 determinations. * P<0.05, ** P<0.01.

Epithelial cell barrier function loss is thought to be the initial inciting event that underlies injury and inflammation in many intestinal disorders, including IBD [19], [20]. Such barrier defects result in the migration of luminal antigens into the submucosa, exposing lamina propria immune cells to these antigens, eliciting inflammatory response and epithelial injury that characterize these diseases [19], [20]. We studied barrier function in water-drinking mice and DSS treated mice using a FITC-labeled dextran method, as described in *Materials and Methods*. Mice were administered FITC-dextran by gavage, and fluorescence was quantified in the serum at 4 h after the administration of FITC-dextran. As shown in Fig. 5, water-drinking mice showed an FITC-dextran of 0.4283±0.1678 mg of FITC/µg protein. In comparison, there was ∼3-fold increase in FITC-dextran levels in DSS mice in day-5 group (1.72±0.4 mg of FITC/µg protein) compared to control mice, suggesting decreased barrier function in these mice, which is consistent with data reported previously [21], [22].

### DSS induces the production of inflammatory mediators

**Figure 6:**
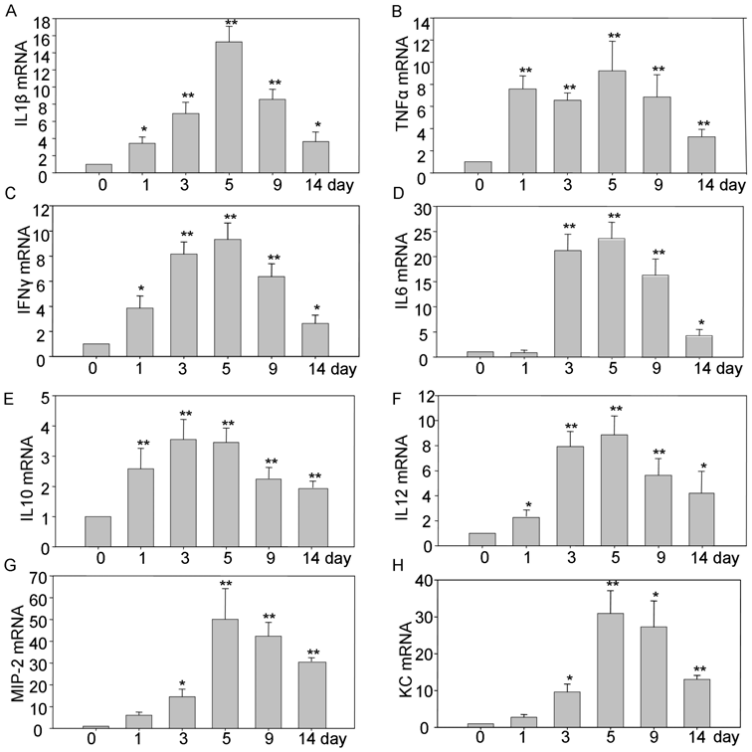
DSS can increase the production significantly of proinflammatory mediators TNF-α, IL-1β, IL-6, IL-10, IL-12, IFN-γ and chemokines KC and MIP-2. This stimulation happened very early with the treatment, TNF-α, IL-1β, IL-10, IL-12, and IFN-γ production increased as early as the first day. The production of these cytokines reached to the peak at the day 5 of DSS treatment, then decreased after the DSS withdrawal, but all of them are still high significantly than those in control mice. * P<0.05, ** P<0.01.

We next considered potential mechanisms which might underlie the colitis exhibited by DSS treatment. Pro-inflammatory mediators play central roles in the pathogenesis of IBD including ulcerative colitis and Crohn's diseases. Enhanced intestinal permeability and consequent immune cell infiltration is thought to increase mucosal production of pro-inflammatory cytokine, both from epithelial cells as well as immune cells. We employed real time PCR to study the proinflammatory cytokines thought to play a pathogeneic role in IBD under DSS treatment in C57BL/6 mice. As shown in Fig. 6, DSS significantly increased the production of proinflammatory cytokines TNF-α, IL-1β, IL-6, IL-10, IL-12, IFN-γ and KC, as well as chemokine MIP-2. Furthermore, this stimulation happened very early after the treatment, in the cases of TNF-α, IL-1β, IFN-γ, IL-10 and IL-12, production increased significantly as early as the first day of treatment. The production of these proinflammatory cytokines or chemokines increased progressively during DSS treatment, reaching a maximum on the 5^th^ day. After DSS withdrawal, these mediators remained at high levels until being sacrificed at day 14, which indicated that the duration of inflammation incited by DSS lasts a very long time, which implied the potential to turn into chronic inflammation.

### DSS induces increases the productions of inflammatory mediators in both proximal and distal parts of colon

**Figure 7:**
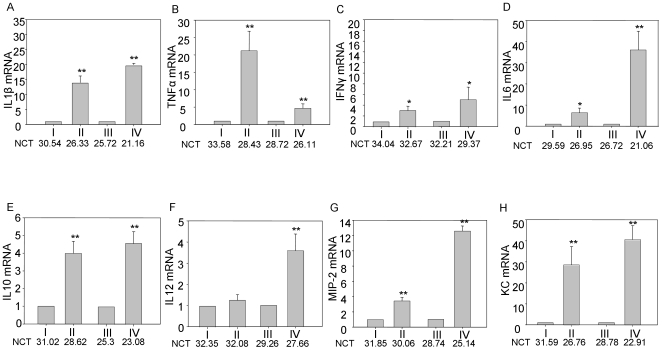
5 days of DSS treatment stimulates the production of proinflammatory cytokines differently in proximal and distal parts of colon. After DSS treatment, the production of cytokines TNF-α, IL-1β, IL-6, IL-10, IL-12, IFN-γ and chemokines KC and MIP-2 increases both in proximal and distal parts of colon. But the increasing folds of TNF-α is higher in proximal colon than that in distal colon, the increasing folds of IL-1β, IL-10 and KC are almost same in both proximal and distal colon. There are higher folds increases in distal colon than in proximal colon for IL-6, IL-12, IFN-γ and chemokine MIP-2. But the normalized cycles thresholds (NCT) are always lower in distal colon than in proximal colon which means higher absolute production of these mediators in distal part than in proximal parts of the colon. I: Water drinking mice proximal colon, II: DSS treated mice proximal colon, III: Water drinking mice distal colon, IV: DSS treated mice distal colon. * P<0.05, ** P<0.01.

In order to understand the cytokine expression in histologically affected distal colon compared to relatively uninvolved proximal colon, we performed real time PCR of pro-inflammatory cytokines for samples from proximal and distal colon. As shown in Fig. 7, with the DSS treatment, the expression of proinflammatory cytokines IL-1β, TNF-α, IFN-γ, IL-6, IL-10, IL-12 and chemokine KC and MIP-2 increase significantly in comparison in samples from control mice both in the proximal and distal colon. Although the absolute increases of these cytokines mRNA were higher in the distal colon than those for the samples from proximal colon, especially IL-1β and IL-6 production made ∼30-fold increase, as reported previously [23]; MIP-2 and IFN-γ increase around 20 times of their control levels; IL-10 and IL-12 expression were enhanced up to 4 times, the proximal colon demonstrated significant increases in these cytokines. IL-6 mRNA increases more than 10 times, IL-1β and IFN-γ increase 5 times of control levels; however, IL-10 and MIP-2 only demonstrated two-fold increases of their production, but no increase IL-12 transcript was detected. TNF-a showed dramatic fold increase in the proximal colon compared to distal colon although the absolute level of this cytokine was higher in the distal colon (Fig. 7).

## Discussion

This work is the second comparitive study carried out since the first paper in 1993 from Cooper et al [17]. In addition to the clinical and histological parameters of colitis described in the original study, our study includes detailed analysis of cytokine profile in a spatial and temporal fashion during the colitic and recovery phase in distal and proximal colon. The clinical and histological changes were determined based on the phenotypic and pathologic changes [17], [24], [25], such as diarrhea, rectal bleeding, body weight loss and colon shortening, which were the common phenomenon seen in DSS-induced experimental colitis. Diarrhea is due to the increased permeability of intestinal cells or hyperosmolarity in lumen led by DSS [26], [27], [28]. Weight loss and the shortening of the colon, as indicators for the severity of intestinal inflammation, correlate with the pathologic and histological changes and are consistent markers for colitis [13], [24], [25]. As a biochemical indicator for intestinal inflammation, the assessment of MPO activity is to quantify intestinal inflammation to assess the tissue damage and extent of infiltration by inflammatory cells [29].

In addition, there is enhanced inflammatory mediators production in DSS treated mouse colon during the colitic phase. Proinflammatory cytokines are local inflammatory mediators, produced by macrophages, lymphocytes as well as by epithelial and mesenchymal cells, involved in the development and pathogenesis of inflammation and immunity [30], [31]. We found that proximal and distal colons express proinflammatory mediators at different levels. In control mice, the basal levels of almost all the cytokines tested are much lower in the proximal colon than those in distal colon. In general, after DSS treatment, the distal colon produced much higher levels of proinflammatory mediators in terms of both folds and absolute value, which suggested that the distal colon is more affected by DSS, which is consistent with data published recently [32]. This is in keeping with the severe histological damage in the distal colon compared to the proximal colon. An exception was the relative increasing folds of TNF-a, which was higher in the proximal colon compared to the distal colon. As expected, the levels of these cytokines return to baseline during the recovery phase. The mechanisms by which DSS induces stronger mucosal inflammation in the distal colon than in proximal colon are not fully understood, but most *in vivo* and *in vitro* studies suggest that DSS causes colitis by interfering directly with intestinal epithelial cell barrier function including crypt damage [17], [33], which is the primary event that leads to secondary mucosal inflammatory responses characterized by both Th1 and Th2 cytokine profiles. These proinflammatory cytokines not only play a role in the pathogenesis of DSS induced colitis [34], but are important as intervention targets against colitis.

In conclusion, we present a rigorous analysis of DSS-induced colitis, a commonly used animal model of IBD, correlating cytokine profile with clinical and histological parameters as well as barrier properties. Together, our data provide novel insight regarding differential expression of cytokines, particularly TNF-α and IL-6, in the proximal and distal colon rendering this model as a useful tool to dissect the role of these cytokines in the induction of inflammation and recovery from it.

## Materials and Methods

### Mouse colitis model

All experiments were carried out in C57BL/6 mice (8 wk, 18–22 g) obtained from Jackson Laboratories (Bar Harbor, ME). Mice were group housed under a controlled temperature (25°C) and photoperiod (12:12-h light-dark cycle) and allowed unrestricted access to standard mouse chow and tap water. They were allowed to acclimate to these conditions for at least 7 days before inclusion in the experiments. Colitis was induced by the addition of DSS [40,000 Da, 3.5% (wt/vol), ICN Biochemicals, Aurora, OH] to the drinking water. The mean DSS-water consumption was recorded for each group. Groups of mice (*n* = 6 mice/group) were treated with 3.5% DSS or regular water for the indicated days. Body weights were assessed every day during the treatment period. Histological assessment of colitis was performed by H&E staining and analyzed by microscopy. All animal experiments were approved by The Animal Care Committee of Emory University, Atlanta and were in accordance with the guide for the Care and Use of Laboratory Animal, published by the U.S. Public Health Service.

### Clinical activity

Colitis was quantified with clinical activity, as described previously [17] using the parameters of weight loss, stool consistency, and fecal blood which were determined daily for each mouse. Five days after the induction of colitis, mice were euthanized by CO2/hypothermia. The abdominal cavity was exposed by a midline laparotomy, and the entire colon was removed from the caecum to the anus. The length of the colon was measured, the colon was flushed with cold PBS and opened longitudinally for morphologic studies, and tissue obtained from each colon was processed for further assays.

### Histological assessment of colitis

Specimens from proximal and distal parts of colon were stained with hematoxylin, histological features were analyzed for these microscopic sections as described previously [17], according to the severity of the induced damage.

### Myeloperoxidase (MPO) activity

Neutrophil infiltration into colon was quantified by measuring MPO activity [35], [36]. Briefly, a portion of colon was homogenized in 1∶20 (w/v) of 50 mM phosphate buffer (pH 6.0) containing 0.5% hexadecyltrimethyl ammonium bromide (Sigma-Aldrich, ST. Louis, MO) on ice using a Polytron homogenizer. The homogenate was sonicated for 10 s, Freeze-thawed three times, and centrifuged at 14,000 rpm for 15 min. Supernatant (14 µl) was added to 1 mg/ml o-dianisidine hydrochloride (Sigma-Aldrich, ST. Louis, MO) and 0.0005% hydrogen peroxide, and the change in absorbance at 460 nm was measured. One unit of MPO activity was defined as the amount that degraded 1 µmol peroxidase per minute at 25°C. The results were expressed as absorbance per gram of tissue.

### *In vivo* permeability assay

In vivo permeability assay to assess barrier function was performed using an FITC-labeled dextran method, as described [37]. Briefly, 8- to 10-wk-old mice were used. Food and water were withdrawn for 4 h and mice were gavaged with permeability tracer (60 mg/100 g body weight of FITC-labeled dextran, MW 4000; Sigma-Aldrich). Serum was collected retro-orbitally; fluorescence intensity of each sample was measured (excitation, 492 nm; emission, 525 nm; Cytofluor 2300; Millipore, Waters Chromatography); and FITC-dextran concentrations were determined from standard curves generated by serial dilution of FITC-dextran. Permeability was calculated by linear regression of sample fluorescence (Excel 5.0; Microsoft).

### Real time PCR

Colons were removed from euthanized mice, washed to remove fecal matter and then divided three of each group into sections corresponding to proximal colon (caecum to mid-transverse colon) and distal colon (mid-transverse colon to anus). Total RNA was extracted using TRIzol (Invitrogen, Carlsbad, CA) from colon mucosa from whole or parts of colon of DSS-treated mice and then reverse transcribed using the Thermoscript™ RT-PCR System (Invitrogen, Carlsbad, CA) and purified with the RNeasy Mini Kit (Qiagen, Germantown, MD). Real time PCR was performed using iQ SYBR Green Supermix kit (BioRad, Hercules, CA) with the iCycler sequence detection system (BioRad, Hercules, CA). Specific primers were designed using the Primer Express Program (Applied Biosystems, Foster City, CA): TNF-α, IL-1, IL-6, KC, IFN-γ, IL-10 IL-12 and MIP-2, 36B4 acts as internal control (Table 1). For graphical representation of quantitative PCR data, raw cycle threshold values (Ct values) obtained for target samples were deducted from the Ct value obtained for internal control transcript levels, using the ΔΔCt method as follows: ΔΔC_T_ = (*C*_t_,_target_−*C*_t_,_con_)_treatment_−(*C*t,_target_−*C*_t_,_con_)_non-treatment_, and the final data were derived from 2^−ΔΔCT^.

**Table 1:** Primers used for proinflammatory mediators quantification.

Name	Nucleotide sequence
34B4For	5′ TCCAGGCTTTGGGCATCA 3′
36B4 Rev	5′ CTTTATCAGCTGCACATCACTCAGA-3′
TNF-α For	5′ AGGCTGCCCCGACTACGT 3′
TNF-α Rev	5′ GACTTTCTCCTGGTATGAGATAGCAAA 3′
IFN-γ For	5′ CAGCAACAGCAAGGCGAAA3′
IFN-γ Rev	5′ CTGGACCTGTGGGTTGTTGAC 3′
IL-1β For	5′ TCG CTCAGGGTCACAAGAAA 3′
IL-1β Rev	5′ CATCAGAGGCAAGGAGGAAAAC 3′
IL-6 For	5′ ACAAGTCGGAGGCTTAATTACACAT 3′
IL-6 Rev	5′ TTGCCATTGCACAACTCTTTTC 3′
KC For	5′ CTTGAAGGTGTTGCCCTCAG 3′
KC Rev	5′ TGGGGACACCTTTTAGCATC 3′
IL-10 For	5′ GGTTGCCAAGCCTTATCGGA 3′
IL-10 Rev	5′ ACCTGCTCCACTGCCTTGCT 3′
IL-12 For	5′ AGACCCTGCCCATTGAACTG 3′
IL-12 Rev	5′ GAAGCTGGTGCTGTAGTTCTCATATTT 3′
MIP-2 For	5′ GGCAAGGCTAACTGACCTGGAAAGG 3′
MIP-2 Rev	5′ ACAGCGAGGCACATCAGGTACGA 3′

Note: TNF-α: Tumor necrosis factor-alpha, IFN-γ: Interferon-gamma, IL-1β: Interleukin-1 beta, IL-6: Interleukin-6, KC: Keratinocyte-derived Chemokine, MIP-2: macrophage inflammatory protein-2, IL-10: Interleukin-10, IL-12: Interleukin-12, For: Forward, Rev: Reverse.
